# Cotton-Tipped Plastic Swabs for SARS-CoV-2 RT-qPCR Diagnosis to Prevent Supply Shortages

**DOI:** 10.3389/fcimb.2020.00356

**Published:** 2020-06-23

**Authors:** Byron Freire-Paspuel, Patricio Vega-Mariño, Alberto Velez, Paulina Castillo, Eliana Elizabeth Gomez-Santos, Marilyn Cruz, Miguel Angel Garcia-Bereguiain

**Affiliations:** ^1^One Health Research Group, Universidad de Las Américas, Quito, Ecuador; ^2^Agencia de Regulación y Control de la Bioseguridad y Cuarentena para Galápagos, Puerto Ayora, Ecuador; ^3^Hospital República del Ecuador, Ministerio de Salud Pública, Puerto Ayora, Ecuador

**Keywords:** SARS-CoV-2, surveillance, swabs, cotton swab, rayon swab

## Abstract

CDC and WHO guidelines for severe acute respiratory syndrome coronavirus 2 (SARS-CoV-2) diagnosis only recommend synthetic fiber swabs for nasopharyngeal (NP) sampling. We show that cotton-tipped plastic swabs do not inhibit PCR and have equivalent performance to rayon swabs. Cotton-tipped plastic swabs are massively produced worldwide and would prevent swab supply shortages under the current high SARS-CoV-2 testing demands, particularly in developing countries.

## Introduction

NP swab is the reference sampling method for SARS CoV2 diagnosis, as recommended by the World Health Organization (WHO) and the Centers for Disease Control and Prevention (CDC) (Center for Diseases Control Prevention, 2020; WHO, 2020a,b). The CDC only endorses the use of synthetic fiber-tipped swabs like rayon or nylon swabs on their recent guidelines for SARS-CoV-2 diagnosis (Center for Diseases Control Prevention, 2020). WHO general guidelines for respiratory sample collection recommend either cotton or synthetic fiber swabs (WHO, 2020b), but recent WHO guidelines for SARS-CoV-2 diagnosis only endorse synthetic fiber swabs (WHO, 2020a).

Multiple *in vitro* RT-qPCR diagnosis kits are available on the market for the detection of SARS-CoV-2. Some of them have received emergency use authorization (EUA) from the U.S. Food & Drug Administration (FDA), while others only report validations made by manufacturers. The CDC-designed 2019-nCoV CDC EUA kit (IDT, USA) is based on the SARS-CoV-2-detecting probes N1 and N2, which have received positive evaluation in recent reports (Nalla et al., 2020; Rhoads et al., 2020), and RNase P as an RNA extraction quality control.

From the beginning of 2020, the COVID-19 pandemic has spread rapidly from Asia to Europe and the USA but also finally to Africa and Latin America. Public health systems have been challenged and have been overwhelmed in developing countries like Ecuador. In this context, the capacity to perform SARS-CoV-2 tests is limited due to a lack of enough laboratory equipment and trained personnel. Moreover, SARS-CoV-2 diagnosis may be disrupted due to supply shortage. For instance, Ecuador is experiencing a supply shortage of synthetic fiber swabs that is causing diagnosis disruption, particularly in isolated locations like the Galapagos Islands, where we implemented the “LabGal” SARS-CoV-2 diagnosis facility. Under this scenario, we conducted a validation study for NP sampling for SARS-CoV-2 diagnosis using easily available cotton-tipped plastic swabs and did not find the inhibition effect on PCR reaction that occurs with those made of wood.

## Methods

### Sample Collection

A total of forty-four (44) subjects suspected of SARS-CoV-2 infection during the surveillance implemented since April 7, 2020 in the Galapagos Islands (Ecuador) were included in the study. All of the subjects were tested for SARS-CoV-2 using two different NP sterile plastic swabs: rayon-tipped swabs and cotton-tipped swabs (Puritan Medical Products LLC, USA; see Supplementary Figure 1). Each NP swab was inserted into the nostril until it hit the back of the NP cavity then rotated five times and removed. The test was conducted in both nostrils for each patient, with <2 min of delay between each sample. NP swabs were immersed in a vial containing 0.5 mL TRIS-EDTA (pH 8) and keep refrigerated until arrival at the lab.

### Viral RNA Extraction and RT-qPCR for SARS-CoV-2

RNA extraction was performed using the PureLink Viral RNA/DNA Mini Kit (Invitrogen, USA) following the manufacturer's instructions. Also, an extraction control (TRIS-EDTA pH 8) was performed for each set of RNA extractions to exclude cross-contamination.

SARS CoV2 was detected using the RT-qPCR CDC protocol. Briefly, two different sets of primers and probes (N1 and N2) are used for SARS-CoV-2 detection, while RNaseP primers and a probe are the housekeeping products for RNA extraction quality control. Following CDC recommendations, the RT-qPCR kit selected was the 2019-nCoV CDC EUA kit (IDT, USA). The assay was validated to detect 1 viral RNA copies/uL by using 2019-nCoV N positive control (IDT, USA) for the N1 and N2 probes. All of the experiments were performed using a CFX96 from BioRad.

### Statistics

For statistical analysis of Ct values, the Student's *t*-test was performed using Excel.

## Results

From the 44 subjects included in the study, 33 (33; 75%) individuals were RT-qPCR SARS-CoV-2 positive and 11 (11; 25%) were negative, either with plastic rayon-tipped or plastic cotton-tipped swabs (Table 1). Taking plastic rayon-tipped swab NP sampling as the gold standard, the detection of SARS-CoV-2 by plastic cotton-tipped swab NP sampling yielded a 100% sensitivity and specificity, indicating total agreement among swabs.

**Table 1:** Performance of plastic cotton-tipped swabs and plastic rayon-tipped swabs for NP sampling for SARS-CoV-2 RT-qPCR diagnosis.

	**Cotton swab SARS CoV-2 positive**	**Cotton swab SARS CoV-2 negative**
Rayon swab SARS CoV-2 positive	33	0
Rayon swab SARS CoV-2 negative	0	11

Ct (mean ± SD) values for N1, N2, and RNaseP amplicons for plastic rayon-tipped swabs (N1: 33.71 ± 3.93; N2: 36.84 ± 3.17; RNaseP: 33.75 ± 3.05) and plastic cotton-tipped swabs (N1: 32.55 ± 5.14; N2: 34.37 ± 5.25; RNaseP: 27.66 ± 2.95) were not statistically different for viral-specific amplicons N1 and N2 (*p* = 0.30 and 0.052, respectively) but were statistically significant (*p* < 0.001) for the RNA extraction quality control housekeeping gene RNaseP, indicating a better RNA extraction yield for plastic cotton-tipped swabs (Table 2).

**Table 2:** RT-qPCR Ct values for N1, N2, and RNaseP probes for nasopharyngeal samples with cotton and rayon swabs (mean +/– SD).

**N**	**Sample**	**N1 Ct**		**N2 Ct**		**RNaseP Ct**	
		**Cotton swab**	**Rayon swab**	**Cotton swab**	**Rayon swab**	**Cotton swab**	**Rayon swab**
1	OCOL	21,11	26,42	26,48	33,2	25,1	30,66
2	ELCA	NA	NA	NA	NA	25,37	29,3
3	MAPI	NA	NA	NA	NA	27,6	26,09
4	CEMI	34,17	34,27	>40	39,94	28,47	29,84
5	460	NA	NA	NA	NA	28,02	34,41
6	462	NA	NA	NA	NA	27,31	33,91
7	465	NA	NA	NA	NA	29,64	33,32
8	467	NA	NA	NA	NA	33,57	36,12
9	471	NA	NA	NA	NA	32,92	34,24
10	474	NA	NA	NA	NA	29,36	36,84
11	943	30,34	32,41	33,23	36,46	25,32	31,43
12	944	31,75	37,41	34,4	>40	21,73	29,76
13	945	32,24	35,05	34,87	>40	23,17	30,8
14	946	37,53	35,8	39,62	40,00	25,27	30,2
15	947	38,1	34,99	>40	>40	23,11	31,45
16	949	27,31	34,95	29,82	>40	25,43	36,68
17	950	23,34	37,31	25,16	>40	22,69	34,6
18	952	38,58	34,65	40,00	39,77	27,74	35,53
19	954	38,12	37,19	>40	>40	26,6	35,73
20	955	35,33	34,6	37,47	40,00	26,37	33
21	963	30,42	32,66	32,37	38,06	29,23	34,64
22	965	31,62	26,7	33,76	32,92	27,32	35,75
23	966	25,53	32,76	28,02	37,49	26,11	27,44
24	967	26,05	31,34	27,94	36,15	26,12	32,29
25	968	23,02	27,3	24,8	31,96	25,04	33,02
26	970	36,01	36,26	40,00	>40	24,87	36,41
27	977	30,37	30,4	33,3	35,31	25,29	29,14
28	978	NA	NA	NA	NA	25,6	37,18
20	979	38,2	38,44	>40	>40	26,97	38,23
30	980	NA	NA	NA	NA	27,77	36,45
31	986	33,9	32,31	37,34	37,12	26,26	36,02
32	987	37,15	>40	37,96	>40	28,84	35,53
33	988	34,76	35,59	36,8	39,27	32,08	34,22
34	989	35,81	36,36	37,05	>40	29,83	36,36
35	990	39,75	37,6	>40	>40	31,55	35,62
36	991	28,8	33,51	29,98	38,74	28,39	38,2
37	992	36,45	26,06	>40	30,08	28,55	30,46
38	993	36,1	39,58	40,00	>40	28,67	34,78
39	996	38,33	38,63	>40	>40	28,86	36,71
40	997	28,27	33,11	29,82	38,4	30,58	38,3
41	999	36,94	29,86	>40	34,04	36,37	35,83
42	1.008	28,11	26,86	29,54	32,46	28,98	35,99
43	1.009	30,64	31,9	32,08	35	30,05	31,11
44	1.010	NA	NA	NA	NA	29,02	31,47

*NA means “not amplified”*.

## Discussion

We herein report that molecular detection of SARS-CoV-2 using plastic cotton-tipped swab NP sampling is as reliable as using plastic swabs tipped with synthetic fibers like rayon, which are considered to be the gold standard by CDC (Center for Diseases Control Prevention, 2020). The main limitation of the study is the relatively small sample size, which would explain the 100% agreement among swabs. However, we believe that a potential disagreement among swabs in a study with a large sample size would be related to variability associated with the sampling procedure more than with the type of swabs. While our results show that cotton does not inhibit the detection of SARS-COV-2, previous work has shown inhibition by the chemicals in the wood stem of some swabs. This may explain why inexpensive cotton swabs have been excluded from CDC and WHO guidelines for SARS-CoV-2 diagnosis (Center for Diseases Control Prevention, 2020; WHO, 2020a). However, the use of cotton-tipped swabs for respiratory specimen collection is included in the WHO's general guidelines for respiratory specimen collection (WHO, 2020b), and it has been reported to be reliable for respiratory retroviruses like influenza specifically (Moore et al., 2008).

Plastic cotton-tipped swabs are cheap and are made worldwide, even in developing countries like Ecuador. Including this type of swab in international guidelines upon more independent validation studies would help to prevent SARS-CoV-2 diagnosis disruption due to swab supply shortage, as recently happened in Ecuador, while keeping high standards for sensitivity and specificity.

To our knowledge, this is the second study comparing swabs for SARS-CoV-2 testing (Vermeiren et al., 2020) but the first study suggesting that inexpensive, readily available cotton swabs could serve as a practical alternative to more costly, imported rayon swabs. Additionally, high sensitivity was recently reported for nasal vs. NP sampling for SARS-CoV-2 diagnosis (Péré et al., 2020). Taking together this finding and ours, even sterile short plastic cotton-tipped swabs like the ones used for ear hygiene could represent an alternative under a lack of NP swab supply. We call upon the worldwide microbiology community, particularly at developing countries, to consider those findings and perform more validation studies to endorse plastic cotton swabs for SARS-CoV2 diagnosis to enhance the testing capacity to fight the spread of the current COVID-19 pandemic.

## Data Availability Statement

The raw data supporting the conclusions of this article will be made available by the authors, without undue reservation.

## Ethics Statement

All samples have been submitted for routine patient care and diagnostics. Ethical approval for this study was not required since all activities are according to legal provisions defined by the “Comité de Operaciones Especiales Regional de Galápagos” that is leading the Covid19 surveillance in Galapagos Islands. No extra specimens were specifically collected for this validation study. All data used in the current study was anonymized prior to being obtained by the authors.

## Author Contributions

MG-B and BF-P wrote the manuscript. All authors contributed to study design, experimental procedures, and revised and approved the final version of the mansucript.

## Conflict of Interest

The authors declare that the research was conducted in the absence of any commercial or financial relationships that could be construed as a potential conflict of interest.
