# Supplementary figures and images for: Cotton-Tipped Plastic Swabs for SARS-CoV-2 RT-qPCR Diagnosis to Prevent Supply Shortages
Source: Front Cell Infect Microbiol. 2020 Jun 23;10:356. doi: 10.3389/fcimb.2020.00356 (PMC7324669; doi:10.3389/fcimb.2020.00356)

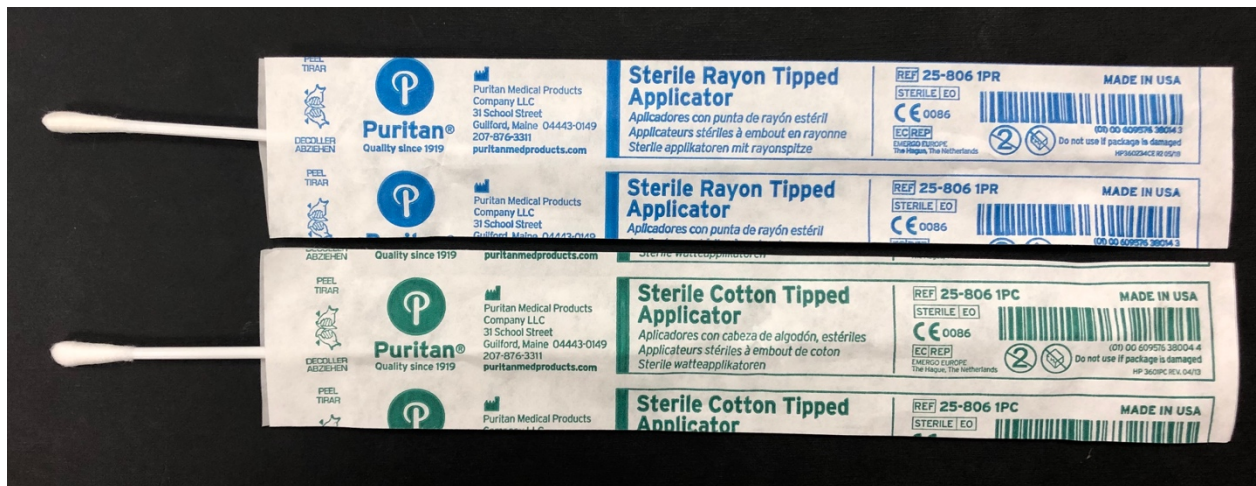

Supplementary Figure S1. Cotton- and Rayon-tipped plastic swabs used in the study.

Supplement: Supplementary file 1 [file Image_1.pdf]
